# Downregulation of the DNA Repair Gene DDB2 by Arecoline Is through p53’s DNA-Binding Domain and Is Correlated with Poor Outcome of Head and Neck Cancer Patients with Betel Quid Consumption

**DOI:** 10.3390/cancers12082053

**Published:** 2020-07-25

**Authors:** Yu-Chu Wang, Jau-Ling Huang, Ka-Wo Lee, Hsing-Han Lu, Yuan-Jen Lin, Long-Fong Chen, Chung-Sheng Wang, Yun-Chiao Cheng, Zih-Ting Zeng, Pei-Yi Chu, Chang-Shen Lin

**Affiliations:** 1 Graduate Institute of Medicine, College of Medicine, Kaohsiung Medical University, Kaohsiung 807, Taiwan; ycwang0214@gmail.com (Y.-C.W.); unrealhank@gmail.com (H.-H.L.); joy11139@yahoo.com.tw (Y.-J.L.); qoxoruby@gmail.com (L.-F.C.); t80467@hotmail.com (C.-S.W.); 2 Department of Bioscience Technology, College of Health Science, Chang Jung Christian University, Tainan 711, Taiwan; jaulingh@mail.cjcu.edu.tw (J.-L.H.); jo286552@hotmail.com (Y.-C.C.); winny3711@gmail.com (Z.-T.Z.); 3 Department of Otorhinolaryngology, Kaohsiung Municipal Ta-Tung Hospital, Kaohsiung 801, Taiwan; kawolee@kmu.edu.tw; 4 Department of Pathology and Medical Research, Show Chwan Memorial Hospital, Changhua 500, Taiwan; chu.peiyi@msa.hinet.net; 5 Center for Cancer Research, Kaohsiung Medical University, Kaohsiung 807, Taiwan; 6 Department of Medical Research, Kaohsiung Medical University Hospital, Kaohsiung Medical University, Kaohsiung 807, Taiwan; 7 Department of Biological Sciences, National Sun Yat-sen University, Kaohsiung 804, Taiwan

**Keywords:** arecoline, betel quid, DDB2, DNA repair, head and neck cancer, p53

## Abstract

Arecoline is the principal alkaloid in the areca nut, a component of betel quids (BQs), which are carcinogenic to humans. Epidemiological studies indicate that BQ-chewing contributes to the occurrence of head and neck cancer (HNC). Previously, we have reported that arecoline (0.3 mM) is able to inhibit DNA repair in a p53-dependent pathway, but the underlying mechanism is unclear. Here we demonstrated that arecoline suppressed the expression of DDB2, which is transcriptionally regulated by p53 and is required for nucleotide excision repair (NER). Ectopic expression of DDB2 restored NER activity in arecoline-treated cells, suggesting that DDB2 downregulation was critical for arecoline-mediated NER inhibition. Mechanistically, arecoline inhibited p53-induced *DDB2* promoter activity through the DNA-binding but not the transactivation domain of p53. Both NER and *DDB2* promoter activities declined in the chronic arecoline-exposed cells, which were consistent with the downregulated *DDB2* mRNA in BQ-associated HNC specimens, but not in those of The Cancer Genome Atlas (TCGA) cohort (no BQ exposure). Lower *DDB2* mRNA expression was correlated with a poor outcome in HNC patients. These data uncover one of mechanisms underlying arecoline-mediated carcinogenicity through inhibiting p53-regulated DDB2 expression and DNA repair.

## 1. Introduction

The areca nut is the fruit of the palm *Areca catechu* and is the basic component in all forms of betel quid (BQ) across different geographic areas. The International Agency for Research on Cancer (IARC) has recognized that areca nut and BQ without tobacco are carcinogenic to humans (Group 1) [1]. There are more than 600 million people who have BQ-chewing habits in the world [1]. Most BQ chewers live in southern and southeast Asia, the South Pacific islands, and some chewers can be found in immigrant communities from these regions. In addition to traditional culture and religion, the psychoactive effect of areca nut is believed to be another cause for the habit of BQ-chewing [2,3], which has led to the areca nut becoming the fourth most common addictive substance, following cigarettes, alcohol, and caffeine [1]. The association between human malignancy and BQ-chewing has been demonstrated for cancers of the head and neck [4,5], esophagus [6], liver [7], and others [8,9]. Notably, an epidemiological study showed that the risk for the development of oral cancer is higher in people with a habit of BQ-chewing (28-fold versus control) than in those with a habit of cigarette (18-fold) or alcohol (10-fold) consumption [4]. This result emphasizes the high carcinogenicity of ingredients of BQs.

Arecoline is the major alkaloid in the areca nut and contributes to the genotoxic effects of the areca nut [10,11,12]. Both arecoline and areca nut extract (ANE) can induce DNA damage, unscheduled DNA synthesis, chromosome abnormality, and micronucleus formation in human epithelial cells and mouse bone-marrow cells [11,12,13,14,15,16]. Some studies suggest that arecoline and ANE can increase reactive oxidative species and induce DNA damage [17,18,19]. Previously, we have reported that arecoline at physiological concentration (0.3 mM) and ANE inhibit DNA repair through the pathways of nucleotide excision repair (NER) and DNA double-strand break (DSB) repair [20,21]. Arecoline and ANE upregulate the expression of *miR-23a*, which targets to the 3′-untranslational region of *FANCG* mRNA, leading to the downregulation of FANCG protein level and impairment of DSB repair [21]. We also showed that arecoline deregulates chromosome segregation and increases the expression of aurora A [22], which is an oncogenic kinase and is overexpressed in many human cancers [23]. In addition, arecoline enhances the mutagenicity of benzo[a]pyrene, which is demonstrated by the comet assay [24]. The inhibitory role of arecoline in NER occurred, at least in part, through interfering with the transactivation function of p53, which results in a decreased expression of *CDKN1A*/*p21^Cip1^*, a representative p53 target gene [20]; however, the mechanism underlying arecoline-mediated inhibition of p53’s function on NER is unclear.

Several studies have shown that p53 can enhance NER through its transactivation activity [25,26]. Upon UV irradiation, p53 targets the promoters of *DDB2* and *XPC*, both of which are NER core factors, and transactivates their expression in human cells [27,28,29,30]. However, p53 cannot activate DDB2 expression in mouse cells because mouse *Ddb2* promoter lacks a functional p53 response element [29]. The role of DDB2 in NER is mediated by its chromatin decondensation activity. DDB2 functions as the subtract adaptor of the DDB1-CUL4 E3 ligase complex, which ubiquitinates histone H2A, H3, and H4 at DNA lesions and facilitates chromatin relaxation, allowing access for DNA repair factors, such as XPC, to DNA lesions [31,32,33]. Individuals with xeroderma pigmentosum syndrome are deficient in the core NER genes, such as *DDB2* and *XPC* (xeroderma pigmentosum complementation group E and C, respectively), and are susceptible to the development of skin cancer [34]. In animal models, *Ddb2*-deficient mice are also predisposed to UV-induced skin cancer and several other types of cancers [35,36]. These results indicate an essential role of DDB2 in protecting cells from UV-induced carcinogenesis.

In this study, we found that the p53-regulated expression of DDB2 was inhibited by arecoline in human head and neck cancer (HNC) cells. This arecoline-mediated inhibition occurred, specifically, through impeding the function of p53’s DNA-binding domain toward *DDB2* promoter. Ectopic overexpression of DDB2 was able to restore arecoline-mediated inhibition of NER. Decreased NER and *DDB2* promoter activities were also observed in long-term arecoline-treated cells. In addition, *DDB2* mRNA was downregulated in BQ-associated HNC specimens, but not in those without BQ exposure. *DDB2* downregulation was correlated with a poor outcome in HNC patients. These results suggest that arecoline may contribute to HNC development through inhibiting p53-regulated DDB2 expression and NER.

## 2. Results

### 2.1. Arecoline Downregulates the Expression of DDB2

**Figure 1:**
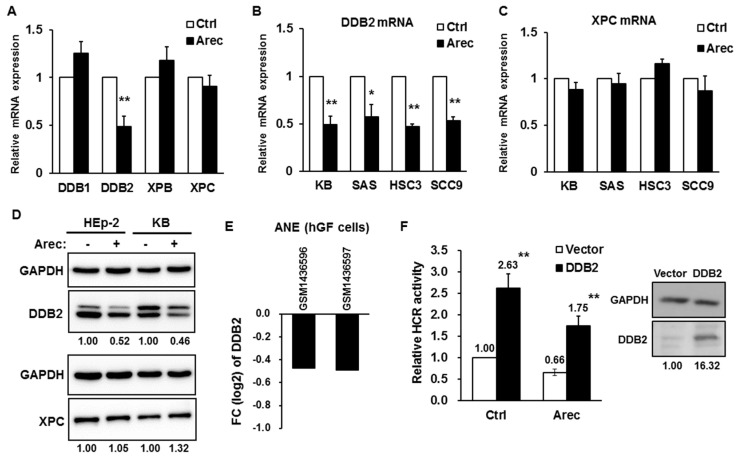
Arecoline specifically downregulates the expression of DDB2. (**A**) RT-qPCR showed that arecoline treatment (0.3 mM, 24 h) decreased *DDB2* mRNA level in HEp-2 cells. The mRNA levels of *DDB1*, *XPB*, and *XPC* were not affected apparently. The relative mRNA expression in vehicle control (H_2_O) was set as one by using GAPDH as an internal control. (**B**) RT-qPCR showed that *DDB2* mRNA level was downregulated in arecoline treated KB, SAS, HSC3, and SCC9 cells. (**C**) The mRNA expression of *XPC* was not affected by arecoline treatment in KB, SAS, HSC3, and SCC9 cells. (**D**) Western blot analyses showed that arecoline treatment (0.3 mM, 24 h) decreased DDB2 protein level in HEp-2 and KB cells. The protein level of XPC was not affected. (**E**) The expression of *DDB2* mRNA was downregulated in ANE-treated human gingival fibroblasts (hGF). The results were extracted from the Gene Expression Omnibus (GSE59414) and the expression level of *DDB2* mRNA was log2 transformed. FC, fold-changed (ANE versus H_2_O). (**F**) Host cell reactivation (HCR) assay showed that overexpression of DDB2 restored arecoline-mediated inhibition of nucleotide excision repair in HEp-2 cells. The expression of flag-tagged DDB2 was detected by Western blot analysis using an anti-flag antibody. All data are shown as mean ± standard deviation (*n* = 3–5). Ctrl, vehicle control (H_2_O); Arec, arecoline. * *p* < 0.05 versus control; ** *p* < 0.01 versus control. The full-length blots for Figure 1D,F can found at Figure S1.

To investigate the mechanism underlying arecoline-mediated inhibition of NER through the p53 pathway, we examined the expression of p53-regulated NER genes *DDB2* and *XPC* upon arecoline treatment with the physiological concentration (0.3 mM for 24 h) by quantitative RT-PCR and Western blot analysis. The results showed that arecoline specifically downregulated the mRNA expression of *DDB2*, but not that of *XPC*, *XPB*, and *DDB1* in HEp-2 cells (Figure 1A). The arecoline-mediated downregulation of *DDB2* mRNA was also observed in other HNC cell lines, such as KB, SAS, HSC3, and SCC9 (Figure 1B); however, the expression of *XPC* mRNA in these HNC cell lines was not affected (Figure 1C). The protein level of DDB2 was decreased in arecoline-treated HEp-2 and KB cells; in contrast, the expression of XPC was not changed (Figure 1D). We also checked the microarray data of ANE-treated human gingival fibroblasts from the Gene Expression Omnibus (GEO, GSE59414) [37] and found that the expression of *DDB2* mRNA was downregulated (Figure 1E). These results suggest that arecoline specifically inhibits DDB2 expression.

### 2.2. Reconstituted DDB2 Expression Restores Arecoline-Inhibited NER Activity

To evaluate whether DDB2 downregulation is critical for arecoline-mediated inhibition of NER [20], we ectopically expressed DDB2 and conducted a host cell reactivation assay to examine the NER activity in arecoline-treated HEp-2 cells. The results showed that overexpression of DDB2 enhanced NER activity in vehicle-control cells (2.63-fold, compared to vector control) and restored arecoline-mediated suppression of NER activity (from 0.66-fold to 1.75-fold, Figure 1F). These data indicate that arecoline inhibits NER, at least in part, through downregulating the expression of DDB2.

### 2.3. Arecoline Inhibits the Recruitment of p53 and RNA Polymerase II to the Promoters of DDB2 and p21^cip1^ in HEp-2 Cells

**Figure 2:**
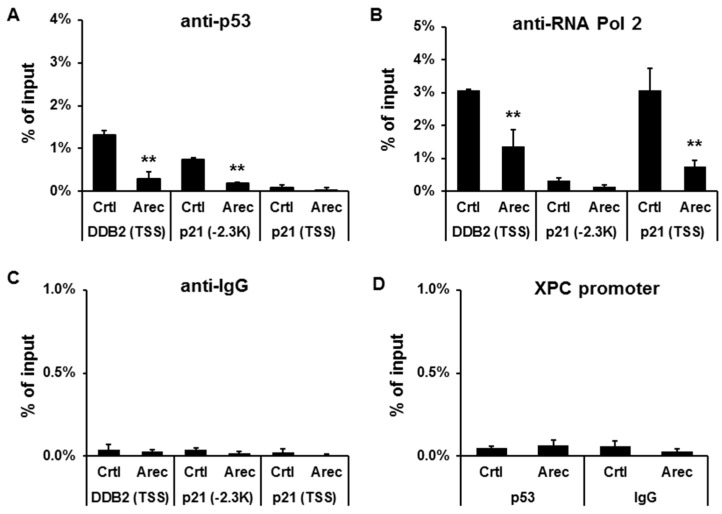
Arecoline inhibits the recruitment of p53 and RNA polymerase II to the promoters of *DDB2* and *p21^Cip1^* (*CDKN1A*) in vivo. The HEp-2 cells were treated with arecoline (0.3 mM) or vehicle (H_2_O) for 24 h and then were harvested for chromatin immunoprecipitation assays using anti-p53 (**A**), anti-RNA polymerase II (**B**), and control IgG (**C**) antibodies followed by quantitative PCR. The PCR amplicons cover the p53-binding sites on the *DDB2* (around the transcription start site, TSS) and *p21^Cip1^* (at 2.3 kilobase upstream to TSS) promoters. (**D**) Chromatin immunoprecipitation assays show no specific binding of p53 and control IgG to the *XPC* promoter. Data are shown as mean ± standard deviation (*n* = 3). Ctrl, H_2_O; Arec, arecoline. ** *p* < 0.01 versus control.

To examine whether arecoline influences p53 binding to the *DDB2* promoter in vivo, a chromatin immunoprecipitation assay, followed by quantitative PCR, was performed. The results showed that the binding of p53 to the promoters of *DDB2* (around the transcription start site, TSS) and *p21^Cip1^* (at 2.3 kilobase upstream to the TSS of *CDKN1A*) was decreased by arecoline treatment (Figure 2A). We have reported that the expression of p21^Cip1^ is suppressed by arecoline in a p53-dependent manner [20]. Meanwhile, the binding of RNA polymerase II to the TSS of *DDB2* and *p21^Cip1^* promoters was also decreased (Figure 2B). No specific binding of control IgG to the promoters of *DDB2, XPC,* and *p21^Cip1^* was detected (Figure 2C,D). We also did not detect a binding of p53 to the *XPC* promoter (Figure 2D). These results suggest that arecoline suppresses DDB2 and p21^Cip1^ expression through inhibiting the recruitment of p53 and RNA polymerase II to their promoters.

### 2.4. Arecoline Inhibits p53-Induced DDB2 Promoter Activity

**Figure 3:**
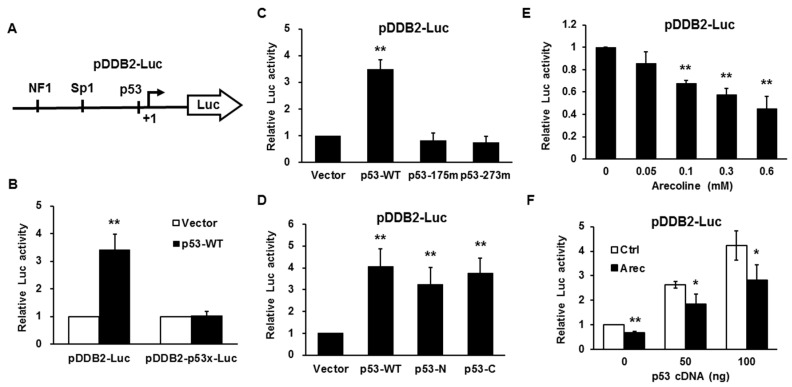
Arecoline inhibits p53-induced *DDB2* promoter activity in HEp-2 cells. (**A**) Schematic illustration of the *DDB2* promoter-luciferase construct (pDDB2-Luc). (**B**) The wild-type p53 (p53-WT) could activate pDDB2-Luc but not pDDB2-p53x-Luc, in which the p53 binding site was mutated. (**C**) The mutations in DNA-binding domain (p53-175m/R175H, p53-273m/R273H) abolished p53-mediated transactivation of pDDB2-Luc. (**D**)The mutations of multiple phosphorylation sites in the N-terminal transactivation domain (p53-N/S6A, S9A, S15A, S18A, S20A, S33A, and S37A) or the C-terminal regulatory domain (p53-C/S315A, S371A, S376A, S378A, and S392A) did not affect p53-mediated transactivation of pDDB2-Luc. (**E**) Arecoline inhibited *DDB2* promoter activity in a dose-dependent manner. (**F**) Overexpression of p53 restored arecoline-mediated inhibition of *DDB2* promoter activity. Data are shown as mean ± standard deviation (*n* = 3). Ctrl, vehicle control (H_2_O); Arec, arecoline; * *p* < 0.05 versus control; ** *p* < 0.01 versus vector (B–D) or control (E,F).

To further investigate the role of p53 in arecoline-mediated inhibition of DDB2 expression, *DDB2* promoter-luciferase reporters containing wild-type (pDDB2-Luc) and mutated (pDDB2-p53x-Luc) p53-binding site were constructed (Figure 3A). Figure 3B shows that ectopic overexpression of p53 increased the activity of wild-type but not that of the mutant *DDB2* promoter, confirming that p53 positively contributed to the expression of DDB2. The roles of p53 functional domains in the activation of the *DDB2* promoter were examined by using various p53 mutant-expression plasmids. The results showed that the p53 with loss-of-function mutations in the DNA-binding (DB) domain (p53-175m/R175H and p53-273m/R273H) abolished its activity of activating the *DDB2* promoter (Figure 3C); however, mutations at multiple phosphorylation sites of the N-terminal transactivation (TA) domain (p53-N/S6A, S9A, S15A, S18A, S20A, S33A, and S37A) or the C-terminal regulatory domain (p53-C/S315A, S371A, S376A, S378A, and S392A) did not affect its ability to activate the *DDB2* promoter (Figure 3D). These results suggest that the DB domain is critical, but phosphorylation at the N- and C-terminal domains is less important for p53-mediated activation of the *DDB2* promoter. Next, we found that arecoline inhibited *DDB2* promoter activity in a dose-dependent manner (Figure 3E) and overexpression of p53 could recover arecoline-mediated inhibition of the *DDB2* promoter (Figure 3F). Together with the decreased binding of p53 to the *DDB2* promoter by arecoline (Figure 2A), these results suggest that p53 is involved in arecoline-mediated inhibition of *DDB2* promoter activity.

### 2.5. Arecoline Inhibits p53-Regulated Promoters through p53’s DNA-Binding Domain

**Figure 4:**
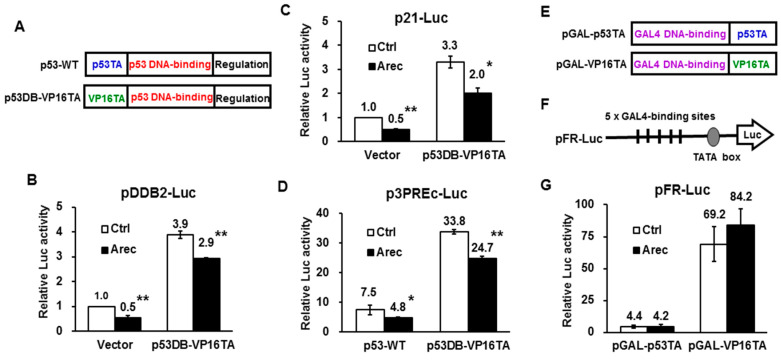
Arecoline inhibits p53-regulated promoters through p53’s DNA-binding domain in HEp-2 cells. (**A**) Schematic diagram shows the DNA-binding (DB) and transactivation (TA) domains of wild-type p53 and the p53DB-VP16TA chimeric construct; (**B–D**) Arecoline (0.3 mM, 24 h) inhibited p53DB-VP16TA-mediated transactivation of the p53 binding site-containing *DDB2* promoter (**B**), *p21^Cip1^* promoter (**C**), and p3PREc-Luc (**D**). The p3PREc-Luc contains only 3 copies of consensus p53-responsive elements and a TATA box [38]. (**E,F**) Schematic illustration of pGAL-p53TA and pGAL-VP16TA chimeric constructs (**E**) and the pFR-Luc reporter that contains 5 copies of GAL4-binding sites (**F**). (**G**) Arecoline (0.3 mM, 24 h) did not inhibit p53’s or VP16’s TA domain-mediated transactivation of pFR-Luc. Data are shown as mean ± standard deviation (*n* = 3). Ctrl, H_2_O; Arec, arecoline; * *p* < 0.05 versus control; ** *p* < 0.01 versus control.

To elucidate the mechanism underlying the repression of p53-induced *DDB2* promoter activity by arecoline, we first focused on the effect of arecoline on p53’s DB domain. To this end, we used the domain-swapped construct, p53DB-VP16TA, in which p53’s TA domain was replaced by VP16’s TA domain (Figure 4A) [26]. When co-transfected with p53-regulated promoters, we found that arecoline repressed p53DB-VP16TA-mediated activation of the promoters of *DDB2* (Figure 4B), *p21^Cip1^* (Figure 4C), and the minimal reporter that contains only three copies of consensus p53-binding sites and a TATA box (p3PREc-Luc, Figure 4D). Next, we analyzed the effect of arecoline on p53’s TA domain specifically. The TA domains of p53 and that of VP16 were fused with GAL4’s DB domain to generate pGAL-p53TA and pGAL-VP16TA, respectively, which could target pFR-Luc, containing five copies of GAL4-binding sites (Figure 4E,F). When co-transfected, we found that arecoline did not suppress the TA domains of p53 and VP16 when they were fused with GAL4’s DB domain (Figure 4G). These results suggest that arecoline suppresses p53-regulated promoters through inhibiting the DB but not the TA domain of p53.

### 2.6. The DDB2 Promoter and NER Activities Are Decreased in Long-Term Arecoline-Treated Cells

**Figure 5:**
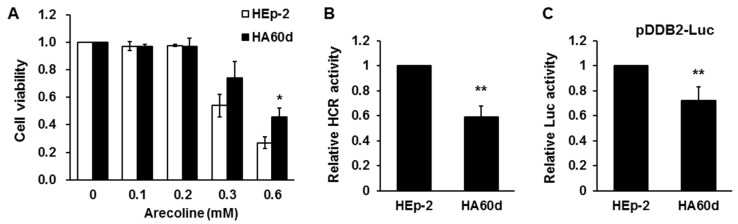
Long-term arecoline treatment leads to suppression of *DDB2* promoter and nucleotide excision repair (NER) activity. The long-term arecoline-treated HA60d cells were obtained by repetitive treatment of arecoline (0.3 mM for 6–8 h/day) for 60 days. (**A**) MTT assays show the cell sensitivity to arecoline treatment for 48 h; (**B**) HCR assay showed an impaired NER activity in HA60d cells; (**C**) *DDB2* promoter activity was decreased in HA60d cells. Data are shown as mean ± standard deviation (*n* = 3–4). *, *p* < 0.05 versus HEp-2 cells; **, *p* < 0.01 versus HEp-2 cells.

Because BQ chewers usually consume BQ daily, we simulated this scenario by repetitively treating the HEp-2 cells with arecoline (0.3 mM, 6–8 h/day) every day for 60 days to obtain HA60d cells, and then examined the activity of the *DDB2* promoter and NER in these cells. Before analysis of the HA60d cells, arecoline was removed from medium for three days; therefore, the obtained results were not from the acute effect (24 h) of arecoline. Instead, the results were more likely to reflect the consequence of long-term arecoline treatment. When compared with parental cells that were cultured in parallel without arecoline treatment, HA60d cells exhibited an increased cell viability in the presence of arecoline for 48 h, as expected (Figure 5A). In addition, HA60d cells exhibited a reduced NER capacity (Figure 5B), consistent with a decreased *DDB2* promoter activity (Figure 5C). These results suggest that long-term BQ chewing may decrease the function of the *DDB2* promoter and NER.

### 2.7. DDB2 mRNA Is Downregulated in Oral Submucous Fibroblasts (OSFs) and HNC in BQ-Epidemic Areas and Is Correlated with Lymph Node Invasion and Patient Outcome

**Figure 6:**
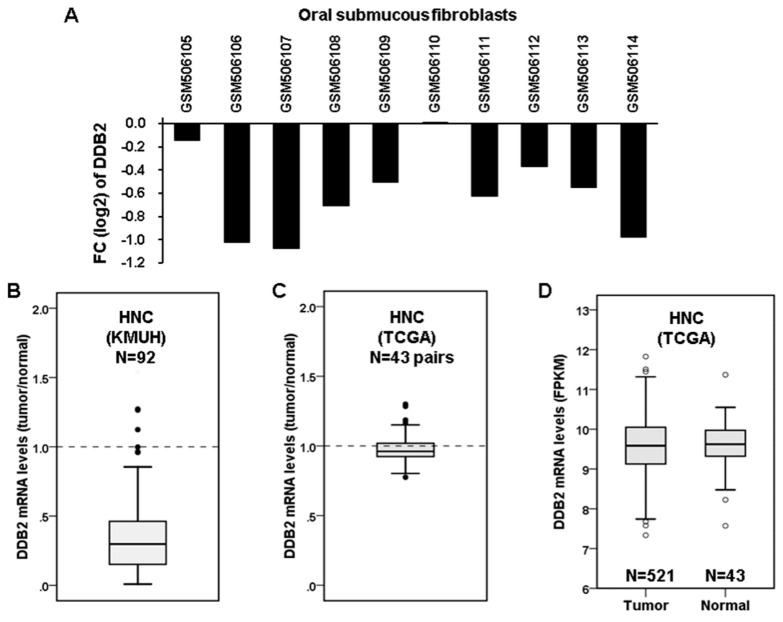
DDB2 is downregulated in oral submucous fibroblasts (OSFs) and head and neck cancer (HNC) specimens collected from betel quid (BQ)-epidemic areas. (**A**) The expression of *DDB2* mRNA was downregulated in 8 out of 10 OSFs in the GSE20170 dataset. FC, fold-changed (OSFs versus normal tissues). (**B**) The *DDB2* mRNA expression in the specimens of BQ-associated HNC versus that in adjacent non-tumor tissues was examined by RT-qPCR and is shown as a ratio in a box plot. The box represents upper and lower quartiles and the horizontal line in the box represents the median expression among the 92 HNC cases of the Kaohsiung Medical University Hospital (KMUH) cohort. (**C,D**) The *DDB2* mRNA expression in the HNC specimens of The Cancer Genome Atlas (TCGA) cohort. The level 3 RNA sequencing data was acquired from TCGA data portal and was checked for the expression of *DDB2* mRNA in (**C**) 43 pairs of tumor/normal samples (shown as a ratio of tumor versus normal) and (**D**) all HNC samples (*n* = 521, shown by fragments per kilobase of transcript per million mapped reads, FPKM).

To examine the role of DDB2 in BQ-associated HNC, we first examined the expression of *DDB2* mRNA in premalignant OSFs, which have high probability to progress to oral cancer, from a BQ-epidemic area using the GEO data set (GSE20170) [39]. The results showed that the expression of *DDB2* mRNA was decreased in 8 out of 10 OSFs (Figure 6A). Next, the expression of *DDB2* mRNA in 92 HNC specimens was compared with that in adjacent non-tumor tissues by using RT-qPCR. The results showed that *DDB2* mRNA was downregulated in most of the BQ-associated HNC cases (Figure 6B). However, the expression of *DDB2* mRNA was not altered in the HNC specimens of The Cancer Genome Atlas (TCGA) cohort (Figure 6C,D), which were collected from HNC patients without a history of BQ-chewing. These results were consistent with the in vitro finding of DDB2 downregulation by arecoline.

**Figure 7:**
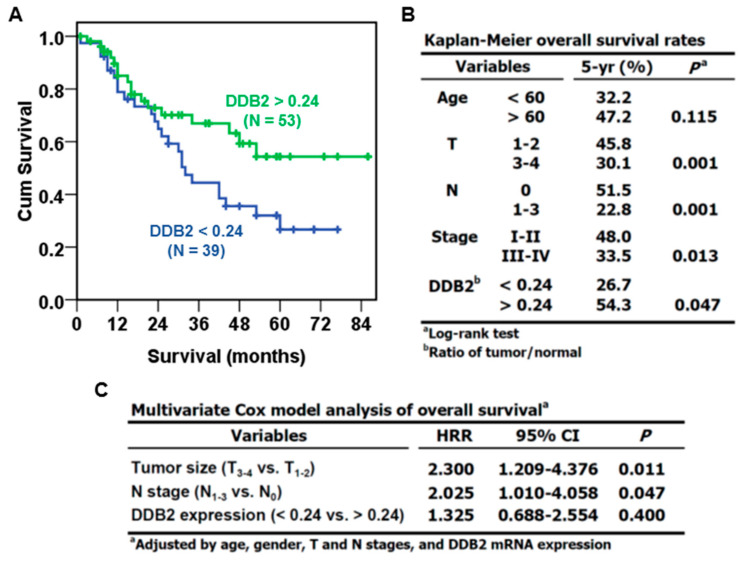
Kaplan–Meier and multivariate Cox regression analyses of overall survival (OS). (**A**) The OS curves of BQ-associated HNC patients (KMUH cohort). (**B**) Patients’ 5-yr OS rate. (**C**) Hazard rate ratio (HRR) of T-, N-stage, and *DDB2* mRNA expression calculated by multivariate Cox model. The patients were sub-grouped based on the expression of *DDB2* mRNA in tumor tissues versus that in adjacent non-tumor tissues (cutoff: 0.24). N: patient number.

The clinical significance of downregulated *DDB2* mRNA was examined by its association with patients’ clinicopathological features. Table 1 shows that the decreased *DDB2* mRNA (ratio of tumor/adjacent non-tumor < 0.24, according to receiver operating characteristic curve analysis) in the HNC of patients with BQ-chewing was positively correlated with lymph node invasion (*p* = 0.007) and death (*p* = 0.002). Kaplan–Meier survival analysis demonstrated that the patients with decreased *DDB2* mRNA levels exhibited poor overall survival (OS) rates (*p* = 0.047, Figure 7A,B). Larger tumor size (*p* = 0.001), positive lymph node involvement (*p* = 0.001), and advanced pathological stage (*p* = 0.013) were also correlated with poor patient outcome (Figure 7B). Multivariate Cox model analysis showed that both T- (HR: 2.300, 95% CI: 1.209–4.376) and N-stage (HR: 2.025, 95% CI: 1.010–4.058) independently predicted worse OS of patients; by contrast, the predictive power of *DDB2* mRNA expression (HR: 1.325, 95% CI: 0.688–2.554) was affected by other confounding factors (Figure 7C).

## 3. Discussion

**Figure 8:**
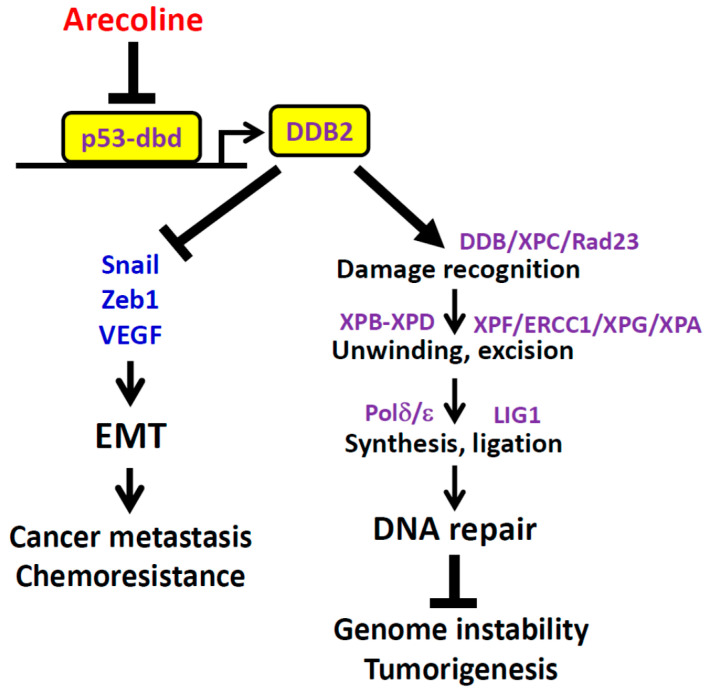
A schematic model of the roles of DDB2 in activating DNA repair and in suppressing epithelial-mesenchymal transition (EMT). DDB2 cooperates with DDB1, XPC, and RAD23 in the recognition of the DNA damage site to initiate the global genome-NER (GG-NER). The following GG-NER steps include DNA unwinding (by XPB and XPD), excision (by XPF, ERCC1, XPG, and XPA), synthesis (by DNA polymerase δ and ε), and ligation (by DNA ligase 1) [34]. This DNA repair function plays a critical role in preventing genome instability and cancer formation [40,41]. DDB2 can also suppress metastasis [65,66] and chemoresistance [67] of cancer cells through inhibiting the expression of EMT activators Sanil, Zeb1, and VEGF. The expression of DDB2 is positively regulated by p53, which binds to the promoter of the *DDB2* gene [28,29]. Arecoline suppresses *DDB2* gene expression through inhibiting p53’s DNA-binding domain (p53-dbd), which may ultimately facilitate tumorigenesis, cancer metastasis, and chemoresistance.

This study demonstrates that the major alkaloid of the areca nut, arecoline, downregulated DDB2 expression through inhibiting p53’s DNA-binding (DB) activity toward the *DDB2* promoter; however, p53’s transactivation (TA) domain was not affected by arecoline. Ectopic expression of DDB2 restored arecoline-inhibited NER activity, suggesting that arecoline-mediated suppression of DDB2 and NER contributes to BQ-induced mutagenicity [10,12,14,15,20]. Because DNA repair serves as an anti-cancer barrier in early human tumorigenesis [40,41], arecoline-induced DDB2 downregulation and impaired NER activity may contribute to cancer development (Figure 8) and, as a result, may lead to a high incidence of HNC among BQ chewers [1,4,5].

Arecoline did not suppress the expressions of other NER genes, such as *DDB1* (Figure 1A), and the expressions of these NER genes were not apparently changed in ANE-treated hGFs and OSFs, except for XPG (*ERCC5*) (Figure S2). XPG is an endonuclease required for excision of damaged DNA during NER [34]. Future work is warranted to investigate the role of XPG in arecoline-mediated suppression of NER.

In addition to the effect of arecoline on the expression of NER genes, whether arecoline influences the post-translational modifications (PTMs) of NER factors is unclear. The PTMs, such as ubiquitylation and SUMOylation, of NER proteins play an important role in the regulation of the NER process [42]. For example, polyubiquitylation of XPC increases its DNA binding affinity to UV-lesions, thus facilitating DNA damage recognition [43]. However, PTMs of DNA repair proteins induced by arecoline or other ingredients of BQ are an unexplored field, and need to be examined in the future to further illustrate the mechanism underlying arecoline-mediated suppression of NER.

In HNC, the function of p53 can be inactivated by gene mutation (the majority in the DB domain) and by the infection of human papillomavirus [44,45,46,47]. Here, we demonstrate another mechanism underlying p53 inactivation in HNC, that is, through arecoline-mediated inhibition of p53’s DB domain. Because p53 plays an important role in the tumorigenesis of HNC [48,49], p53 inactivation through this mechanism may have an important impact on the development of HNC in BQ-epidemic areas, where there are more than 600 million BQ chewers [1].

It is still unclear how arecoline inhibits p53’s DB activity. The diverse functions of p53, such as DNA repair, cell cycle arrest, apoptosis, senescence, and energetic metabolism, can be regulated by PTMs of the p53 protein [50]. Previously, we have shown that arecoline treatment induces hyperphosphorylation at serine 15 (S15-p) of p53’s TA domain [20]. However, S15-p may not be directly involved in arecoline-mediated repression of *DDB2* promoter activity, because arecoline did not affect p53’s TA domain (Figure 4G), and the N-terminal serine mutations did not impair p53-induced *DDB2* promoter activity (Figure 3D). The acetylation at lysine 120 (K120-ac) of p53’s DB domain by the lysine acetyltransferase hMOF or Tip60 regulates p53’s DB activity and contributes to the activation of a subset of p53 target genes [51,52]. Whether arecoline affects p53’s DB domain through regulating K120-ac or other PTMs requires further investigation.

It has been reported that BRCA1 interacts with p53 and enhances p53 binding to the *DDB2* promoter [53,54,55]. Interestingly, both Chiang’s study [56] and our unpublished data show that arecoline treatment resulted in BRCA1 downregulation. In addition, the expression of BRCA1 is decreased in mice with chronic exposure to ANE [57,58]. Therefore, it is of interest to investigate whether BRCA1 downregulation is involved in arecoline-mediated inhibition of p53 binding to the *DDB2* promoter in the future.

In addition to arecoline-mediated repression of p53’s DB domain, other mechanisms are reported to contribute to DDB2 downregulation in HNC. For example, allelic imbalance and loss of heterozygosity at the *DDB2* locus (11p12-11) are observed in some HNC samples [59], suggesting that allelic loss of the *DDB2* gene may lead to DDB2 downregulation in cancer cells. Knijnenburg et al. report an increased methylation at the *DDB2* promoter in a subset of HNC samples [60]. Whether arecoline affects methylation at the *DDB2* promoter is unclear; however, arecoline is reported to increase the recruitment of DNMT3B to the *ANK1* promoter [61]. Long-term arecoline treatment also enhances the expression of DNMT3B, which promotes methylation at the *ALDH1A2* and *ADHFE1* promoters [62]. In this regard, the methylation state of the *DDB2* promoter in the long-term arecoline-treated HA60d cells, as well as in HNC specimens, can be examined in the future.

Downregulation of *DDB2* mRNA was mainly observed in the OSFs and HNC of patients with BQ-chewing history, but not in those without BQ exposure (Figure 6). Furthermore, downregulation of *DDB2* mRNA was correlated with lymph node metastasis and poor overall survival of HNC patients with BQ-chewing habits (Table 1 and Figure 7), although the effect of DDB2 on HNC patients’ survival might be regulated by other confounding factors, especially the status of lymph node involvement (data not shown). The association between downregulated DDB2 expression and poor patient survival is also observed in colorectal cancer [63], astrocytoma [64], and another HNC cohort [65]. The role of DDB2 downregulation in patients’ worse outcomes may be due to its role in suppressing epithelial-to-mesenchymal transition (EMT) [65,66], which is a process involved in metastasis and chemoresistance of cancer cells [67] (Figure 8). Indeed, previous studies show that DDB2 is downregulated in metastatic colorectal and breast cancers [66,68]. Interestingly, arecoline is known to promote EMT [69,70,71]. These results suggest that arecoline-mediated DDB2 downregulation may contribute to EMT and lymph node metastasis of HNC cells. However, this notion needs to be verified further.

## 4. Materials and Methods

### 4.1. Cell Culture and Arecoline Treatment

The human HNC cell lines HEp-2, KB, SAS, HSC3, and SCC9 were grown in Dulbecco’s modified Eagle’s medium (HyClone, Logan, UT, USA) containing 10% fetal bovine serum (Invitrogen, Carlsbad, CA, USA) at 37 °C and 5% CO_2_ with saturating humidity as described previously [20,72]. Arecoline (Sigma-Aldrich, St. Louis, MO, USA) was dissolved in distilled water at 100 mM as a stock and was stored at −20 °C in aliquot. For most experiments, cells were treated with arecoline at 0.3 mM, which is an average concentration in the oral cavity of BQ chewers [73], for 24 h, and then were harvested for subsequent analyses.

### 4.2. Analysis of DNA Repair Activity Using Host Cell Reactivation (HCR) Assay

The HCR assay for NER was conducted as previously described [20,74]. Briefly, the UV (1000 J)-irradiated firefly luciferase reporter pCMV-Luc was co-transfected with pRL-CMV (internal control for calibrating transfection efficiency) using Lipofectamine 2000 (Invitrogen) into cells with or without arecoline treatment for 24 h, which allowed cells to repair the damaged pCMV-Luc. As a result, the firefly luciferase derived from the UV-damaged pCMV-Luc depends on the repair function of the transfected host cells. In parallel, an undamaged pCMV-Luc was also transfected to serve as a reference (100% luciferase activity) for the UV-damaged one. After cell harvest, dual-luciferase assay was conducted and the HCR activity was represented by the ratio of luciferase activity derived from the UV-damaged pCMV-Luc to that derived from the undamaged pCMV-Luc.

### 4.3. Promoter-Luciferase Reporters and DDB2- and p53-Expressing Plasmids

The *DDB2* promoter-luciferase reporter (pDDB2-Luc) was constructed by the nested PCR method to overcome the difficulty in the amplification of the *DDB2* core promoter region that contains multiple CG-repeated sequences. The two pairs of nested PCR primers are the outer primers GTTCGTGTCAGGAAGTCAAGGC, ACAGGCAGTACCGGAGCCCTTC and the inner primers GGGGCTAGCGGGACCATCTTTGCTCCAG, GGGAAGCTTCGCGTCCTCCGTGTGAAG. The nested PCR products that contained the *DDB2* core promoter sequence (−142 to +195) were cloned to the pGL3-basic luciferase reporter (Progema, Ipswich, WI, USA) using NheI and HindIII restriction sites. The deletion of p53-binding site on the pDDB2-Luc was generated by overlapping PCR method using Q5 Site-Direct Mutagenesis Kit (New England BioLabs, MA, USA) and the primers GGGTCGCTTTGGCGGGAAGTTGGCT, AGGGGGAATTCAAACCAGCTTGGAGCTC to obtain pDDB2-p53x-Luc. Both the wild-type and mutant *DDB2* promoter sequences were verified by DNA sequencing. The reporter plasmids p21-Luc (2.4-kb *CDKN1A*/*p21^Cip1^* promoter containing two p53-binding sites) and p3PREc-Luc (3 copies of consensus p53-binding sites and TATA box), as well as the wild-type and mutant p53-expressing plasmids (p53-WT, p53-175m, p53-273m, p53DB-VP16, pGAL4-p53TA, pGAL4-VP16TA), have been described in previous papers [26,38]. The DDB2-expressing plasmid was purchased from OriGene (RC200390, Rockville, MD, USA). The pFR-Luc (5 copies of GAL4-binding sites and TATA box) and pRL-CMV (serve as an internal control for transfection) were from Stratagene (Santa Clara, CA, USA) and Promega, respectively.

### 4.4. Dual-Luciferase Assay

The dual-luciferase assays were performed as described previously [20]. Briefly, cells were co-transfected with 250 ng of various reporters and p53- or DDB2-expressing plasmids (at indicated amounts) using Lipofectamine 2000 (Invitrogen) in the presence of the internal control reporter pRL-CMV (30 ng) for 6 h, then the cells were washed and treated with arecoline for an additional 24 h (or indicated times) and harvested for dual-luciferase assay (Promega) according to manufacturer’s instructions.

### 4.5. Chromatin Immunoprecipitation (ChIP) Assay

ChIP assay was conducted using SimpleChIP Enzymatic Chromatin IP Kit (#9003, Cell Signaling) and the method as described [75] with slight modifications. Briefly, cells were fixed with 1% (*v*/*v*) formaldehyde at room temperature (RT) for 10 min and neutralized with glycine at RT for 5 min. The fixed cells were sonicated using Q700 sonicator (Qsonica, Newtown, CT, USA) to obtain chromatin fragments with a range between 150 and 800 bp. The fragmented chromatin (5 μg) of each treatment was subjected to immunoprecipitation using p53 and RNA polymerase II antibodies (sc-126X and sc-899X, respectively, Santa Cruz), and then were purified for qPCR. A normal immunoglobulin G (sc-2025, Santa Cruz) was used as a negative control. The primer sequences for ChIP-qPCR analyses are: DDB2 (TSS): GCTCCAAGCTGGTTTGAACA and TAGCCGAGCTAAGCCAACTTCC; XPC (TSS): GCCGCGCGTTTCCGAGCC and CGCGGCCGGGTGCGTCAC [30]; p21 (TSS): TATATCAGGGCCGCGCTG and GGCTCCACAAGGAACTGACTTC; and p21 (−2.3 K): AGCAGGCTGTGGCTCTGATT and CAAAATAGCCACCAGCCTCTTCT [76], where TSS represents transcription start site. The amplicons of DDB2 (TSS), XPC (TSS), and p21 (−2.3 K) contain p53-binding regions.

### 4.6. RNA Extraction, Reverse Transcription and Real-Time Quantitative PCR (RT-qPCR)

As described previously [20,74], total RNA was isolated using Tri-reagent (Sigma-Aldrich) and one microgram of total RNA was reverse transcribed to cDNA in a volume of 20 μL using a High-Capacity cDNA Archive Kit (Applied Biosystems, Foster City, CA, USA). The resultant cDNA was diluted to 100 μL with distilled water and 2 μL of diluted cDNA was used for qPCR reaction (20 μL) with PowerSYBR Green reagent (Applied Biosystems) and cycling condition: 50 °C for 2 min, 95 °C for 10 min, followed by 40 cycles at 95 °C for 15 sec and 60 °C for 1 min in the ABI StepOne System. A dissociation (melting) curve analysis was used to check the specificity of qPCR reaction. The relative mRNA expression of *DDB2* (and other genes) in each sample was normalized to that of *GAPDH* and represented by the 2^−ΔΔC^_T_ method. The primer sequences were *DDB1*: CCCCTCAATTCAGATGGCTA and GGTGAGGGTGCTATTGTTGG; *DDB2*: TCAAGGACAAACCCACCTTC and AAACTTCAGCCCAGTGATGC; *XPB*: ACTGGATGGAGCTGCAGAAT and GACATAGGGCACCAGACCTC; *XPC*: AGACCATACCAGAGCCCATTT and TCCATGTGTTTTGCCTGAAA; and *GAPDH*: AGCCACATCGCTCAGACAC and GCCCAATACGACCAAATCC.

### 4.7. Western Blot

Western blot analysis was performed as described [20,74]. Briefly, cell lysates were prepared using RIPA lysis buffer (50 mM Tris-HCl (pH 8.0), 150 mM NaCl, 0.5% (*w*/*v*) sodium deoxycholate, 1% (*v*/*v*) Nonidet P-40, 0.1% (*w*/*v*) SDS, 1 mM DTT) in the presence of protease inhibitors (Roche, Mannheim, Germany). Protein lysates (30 μg) were separated by sodium dodecyl sulfate-polyacrylamide gel electrophoresis, followed by transferring to polyvinylidene difluoride membranes (Millipore, Bedford, MA, USA), incubating with antibodies, and visualizing by enhanced chemiluminescence (Millipore, Bedford, MA, USA) and the ChemiDoc-It imaging system (UVP, Upland, CA, USA). The primary antibodies against DDB2 and XPC were purchased from Cell Signaling (#5416, Danvers, MA, USA) and GeneTex (GTX70294, Irvine, CA, USA), respectively. The antibody against GAPDH (sc-32233, Santa Cruz, CA, USA) was used as a loading control.

### 4.8. Simulation of BQ-Chewing Habit by Long-Term Repetitive Arecoline Treatment

To simulate the habit of BQ chewers, a long-term (up to 60 days) arecoline-treated HEp-2 cell model was established by repetitive arecoline (0.3 mM) on-off treatment daily (6–8 h per day). The resulting cells (HA60d) were cultured without arecoline for at least 3 additional days and then were examined for *DDB2* promoter activity and DNA repair capacity. These results represent the long-term but not acute (24 h) effect of arecoline.

### 4.9. Analysis of DDB2 mRNA Expression in BQ-Associated HNC Specimens

This study protocol (IRB-950094) was approved by the Institutional Review Board of Kaohsiung Medical University Hospital (KMUH) and specimens were collected from 92 HNC patients with BQ-chewing histories after obtaining their written informed consent (KMUH cohort). Among the 92 HNC specimens, 60 had paired adjacent non-tumor tissues. RNA extraction, reverse transcription, and qPCR were conducted for each tumor and non-tumor sample by using *GAPDH* as an internal control, as described [74,77]. For each sample, at least 2 independent RT-qPCR reactions were performed to obtain an average expression level. The expression of *DDB2* mRNA was represented as a ratio of *DDB2* mRNA level in tumors to that in paired non-tumor tissues. For the 32 HNC specimens without paired non-tumor tissues, an average of *DDB2* mRNA level in all non-tumor tissues was used for comparison. The results were shown as a box plot using IBM SPSS Statistics (version 22, Armonk, NY, USA).

### 4.10. Acquisition of HNC Dataset from the Cancer Genome Atlas (TCGA)

TCGA Level 3 RNA-sequencing data of 521 HNC and 43 adjacent normal samples were downloaded from TCGA data portal (https://portal.gdc.cancer.gov/) on 24 February, 2015. The expression of *DDB2* mRNA was represented as fragments per kilobase of transcript per million mapped reads (FPKM) in a box plot, using IBM SPSS Statistics.

### 4.11. Statistical Analysis

For cell culture experiments, data were presented as mean ± standard deviation from at least three independent experiments (as indicated in Figure legends). The difference between control and experimental groups was examined using Student’s *t*-test and *p* < 0.05 was considered statistically significant. For clinical analysis, BQ-associated HNC patients were divided into two groups according to *DDB2* mRNA expression (cutoff of tumor/normal: 0.24) by receiver operating characteristic curve analysis. The overall survival rates were calculated by Kaplan–Meier estimates and log-rank tests. The hazard rate ratio for patients’ overall survival was estimated using the multivariate Cox regression model.

## 5. Conclusions

The present study shows that the major alkaloid of the areca nut, arecoline, inhibited p53’s DNA-binding domain toward the *DDB2* promoter, resulting in downregulation of DDB2 and suppression of NER activity. These findings provide a mechanistic explanation for arecoline- and areca nut-induced genotoxicity. The downregulation of *DDB2* mRNA was observed in BQ-associated OSFs and HNC and was correlated with metastatic lymph node and patients’ worse overall survival rate.

## Figures and Tables

**Table 1:** Correlation between clinicopathological variables and DDB2 mRNA expression.

Variables		DDB2 mRNA ^a^				*p* ^b^
		<0.24		>0.24		
Gender	Male	38	(43.7%)	49	(56.3%)	0.391 ^c^
	Female	1	(20.0%)	4	(80.0%)	
Age	<60	21	(38.9%)	33	(61.1%)	0.418
	>60	18	(47.4%)	20	(52.6%)	
T	1–2	19	(35.8%)	34	(64.2%)	0.139
	3–4	20	(51.3%)	19	(48.7%)	
N	0	18	(31.6%)	39	(68.4%)	0.007
	1–3	21	(60.0%)	14	(40.0%)	
Stage	I–II	12	(30.0%)	28	(70.0%)	0.035
	III–IV	27	(51.9%)	25	(48.1%)	
Death	No	14	(28.0%)	36	(72.0%)	0.002
	Yes	25	(59.5%)	17	(40.5%)	

^a^ Ratio of tumor/normal; cut-off point was determined by the receiver operating characteristic curve. ^b^ Chi-square test. ^c^ Fisher’s exact test.

## Supplementary Materials

The following are available online at https://www.mdpi.com/2072-6694/12/8/2053/s1, Figure S1: The full-length blots for Figure 1D,F, Figure S2: Expression of NER genes in GSE59414, which is shown by an average of 2 samples of areca nut extract (ANE)-treated human gingival fibroblast (hGF), and in GSE20170, which is shown by an average of 10 oral submucous fibroblast (OSF) and standard deviations, FC (log2), fold-changed with log2-transformed.

## References

[1] IARC (2004). Betel-quid and Areca-nut Chewing and Some Areca-nut-derived Nitrosamines-Summary of Data Reported and Evaluation. IARC Monographs on the Evaluation of Carcinogenic Risks to Humans.

[2] Gupta P.C., Warnakulasuriya S. (2002). Global epidemiology of areca nut usage. Addict. Biol..

[3] Winstock A.R., Trivedy C.R., Warnakulasuriya K.A., Peters T.J. (2000). A dependency syndrome related to areca nut use: Some medical and psychological aspects among areca nut users in the Gujarat community in the UK. Addict. Biol..

[4] Ko Y.C., Huang Y.L., Lee C.H., Chen M.J., Lin L.M., Tsai C.C. (1995). Betel quid chewing, cigarette smoking and alcohol consumption related to oral cancer in Taiwan. J. Oral Pathol. Med..

[5] Lee K.W., Kuo W.R., Tsai S.M., Wu D.C., Wang W.M., Fang F.M., Chiang F.Y., Ho K.Y., Wang L.F., Tai C.F. (2005). Different impact from betel quid, alcohol and cigarette: Risk factors for pharyngeal and laryngeal cancer. Int. J. Cancer.

[6] Wu M.T., Lee Y.C., Chen C.J., Yang P.W., Lee C.J., Wu D.C., Hsu H.K., Ho C.K., Kao E.L., Lee J.M. (2001). Risk of betel chewing for oesophageal cancer in Taiwan. Br. J. Cancer.

[7] Wu G.H., Boucher B.J., Chiu Y.H., Liao C.S., Chen T.H. (2009). Impact of chewing betel-nut (Areca catechu) on liver cirrhosis and hepatocellular carcinoma: A population-based study from an area with a high prevalence of hepatitis B and C infections. Public Health Nutr..

[8] Wen C.P., Tsai M.K., Chung W.S., Hsu H.L., Chang Y.C., Chan H.T., Chiang P.H., Cheng T.Y., Tsai S.P. (2010). Cancer risks from betel quid chewing beyond oral cancer: A multiple-site carcinogen when acting with smoking. Cancer Causes Control..

[9] Sharan R.N., Mehrotra R., Choudhury Y., Asotra K. (2012). Association of betel nut with carcinogenesis: Revisit with a clinical perspective. PLoS ONE.

[10] Shirname L.P., Menon M.M., Bhide S.V. (1984). Mutagenicity of betel quid and its ingredients using mammalian test systems. Carcinogenesis.

[11] Stich H.F., Stich W., Lam P.P. (1981). Potentiation of genotoxicity by concurrent application of compounds found in betel quid: Arecoline, eugenol, quercetin, chlorogenic acid and Mn2+. Mutat. Res..

[12] Panigrahi G.B., Rao A.R. (1982). Chromosome-breaking ability of arecoline, a major betel-nut alkaloid, in mouse bone-marrow cells in vivo. Mutat. Res..

[13] Sharan R.N., Wary K.K. (1992). Study of unscheduled DNA synthesis following exposure of human cells to arecoline and extracts of betel nut in vitro. Mutat. Res..

[14] Stich H.F., Stich W. (1982). Chromosome-damaging activity of saliva of betel nut and tobacco chewers. Cancer Lett..

[15] Stich H.F., Stich W., Parida B.B. (1982). Elevated frequency of micronucleated cells in the buccal mucosa of individuals at high risk for oral cancer: Betel quid chewers. Cancer Lett..

[16] Sundqvist K., Liu Y., Nair J., Bartsch H., Arvidson K., Grafstrom R.C. (1989). Cytotoxic and genotoxic effects of areca nut-related compounds in cultured human buccal epithelial cells. Cancer Res..

[17] Shih Y.T., Chen P.S., Wu C.H., Tseng Y.T., Wu Y.C., Lo Y.C. (2010). Arecoline, a major alkaloid of the areca nut, causes neurotoxicity through enhancement of oxidative stress and suppression of the antioxidant protective system. Free Radic. Biol. Med..

[18] Thangjam G.S., Kondaiah P. (2009). Regulation of oxidative-stress responsive genes by arecoline in human keratinocytes. J. Periodontal Res..

[19] Chang M.C., Ho Y.S., Lee P.H., Chan C.P., Lee J.J., Hahn L.J., Wang Y.J., Jeng J.H. (2001). Areca nut extract and arecoline induced the cell cycle arrest but not apoptosis of cultured oral KB epithelial cells: Association of glutathione, reactive oxygen species and mitochondrial membrane potential. Carcinogenesis.

[20] Tsai Y.S., Lee K.W., Huang J.L., Liu Y.S., Juo S.H., Kuo W.R., Chang J.G., Lin C.S., Jong Y.J. (2008). Arecoline, a major alkaloid of areca nut, inhibits p53, represses DNA repair, and triggers DNA damage response in human epithelial cells. Toxicology.

[21] Tsai Y.S., Lin C.S., Chiang S.L., Lee C.H., Lee K.W., Ko Y.C. (2011). Areca nut induces miR-23a and inhibits repair of DNA double-strand breaks by targeting FANCG. Toxicol. Sci..

[22] Wang Y.C., Tsai Y.S., Huang J.L., Lee K.W., Kuo C.C., Wang C.S., Huang A.M., Chang J.Y., Jong Y.J., Lin C.S. (2010). Arecoline arrests cells at prometaphase by deregulating mitotic spindle assembly and spindle assembly checkpoint: Implication for carcinogenesis. Oral Oncol..

[23] Nikonova A.S., Astsaturov I., Serebriiskii I.G., Dunbrack R.L., Golemis E.A. (2013). Aurora A kinase (AURKA) in normal and pathological cell division. Cell. Mol. Life Sci..

[24] Huang J.L., Lu H.H., Lu Y.N., Hung P.S., Lin Y.J., Lin C.C., Yang C.C., Wong T.Y., Lu S.Y., Lin C.S. (2016). Enhancement of the genotoxicity of benzo[a]pyrene by arecoline through suppression of DNA repair in HEp-2 cells. Toxicol. In Vitro.

[25] Wang X.W., Yeh H., Schaeffer L., Roy R., Moncollin V., Egly J.M., Wang Z., Freidberg E.C., Evans M.K., Taffe B.G. (1995). p53 modulation of TFIIH-associated nucleotide excision repair activity. Nat. Genet..

[26] Liu M.T., Chang Y.T., Chen S.C., Chuang Y.C., Chen Y.R., Lin C.S., Chen J.Y. (2005). Epstein-Barr virus latent membrane protein 1 represses p53-mediated DNA repair and transcriptional activity. Oncogene.

[27] Adimoolam S., Ford J.M. (2002). p53 and DNA damage-inducible expression of the xeroderma pigmentosum group C gene. Proc. Natl. Acad. Sci. USA.

[28] Hwang B.J., Ford J.M., Hanawalt P.C., Chu G. (1999). Expression of the p48 xeroderma pigmentosum gene is p53-dependent and is involved in global genomic repair. Proc. Natl. Acad. Sci. USA.

[29] Tan T., Chu G. (2002). p53 binds and activates the xeroderma pigmentosum DDB2 gene in humans but not mice. Mol. Cell. Biol..

[30] Hastak K., Adimoolam S., Trinklein N.D., Myers R.M., Ford J.M. (2012). Identification of a Functional In Vivo p53 Response Element in the Coding Sequence of the Xeroderma Pigmentosum Group C Gene. Genes Cancer.

[31] Wang H., Zhai L., Xu J., Joo H.Y., Jackson S., Erdjument-Bromage H., Tempst P., Xiong Y., Zhang Y. (2006). Histone H3 and H4 ubiquitylation by the CUL4-DDB-ROC1 ubiquitin ligase facilitates cellular response to DNA damage. Mol. Cell.

[32] Lan L., Nakajima S., Kapetanaki M.G., Hsieh C.L., Fagerburg M., Thickman K., Rodriguez-Collazo P., Leuba S.H., Levine A.S., Rapic-Otrin V. (2012). Monoubiquitinated histone H2A destabilizes photolesion-containing nucleosomes with concomitant release of UV-damaged DNA-binding protein E3 ligase. J. Biol. Chem..

[33] Kapetanaki M.G., Guerrero-Santoro J., Bisi D.C., Hsieh C.L., Rapic-Otrin V., Levine A.S. (2006). The DDB1-CUL4ADDB2 ubiquitin ligase is deficient in xeroderma pigmentosum group E and targets histone H2A at UV-damaged DNA sites. Proc. Natl. Acad. Sci. USA.

[34] Sugasawa K. (2008). Xeroderma pigmentosum genes: Functions inside and outside DNA repair. Carcinogenesis.

[35] Yoon T., Chakrabortty A., Franks R., Valli T., Kiyokawa H., Raychaudhuri P. (2005). Tumor-prone phenotype of the DDB2-deficient mice. Oncogene.

[36] Itoh T., Cado D., Kamide R., Linn S. (2004). DDB2 gene disruption leads to skin tumors and resistance to apoptosis after exposure to ultraviolet light but not a chemical carcinogen. Proc. Natl. Acad. Sci. USA.

[37] Pant I., Kumar N., Khan I., Rao S.G., Kondaiah P. (2015). Role of Areca Nut Induced TGF-beta and Epithelial-Mesenchymal Interaction in the Pathogenesis of Oral Submucous Fibrosis. PLoS ONE.

[38] Lin C.S., Kuo H.H., Chen J.Y., Yang C.S., Wang W.B. (2000). Epstein-barr virus nuclear antigen 2 retards cell growth, induces p21(WAF1) expression, and modulates p53 activity post-translationally. J. Mol. Biol..

[39] Khan I., Agarwal P., Thangjam G.S., Radhesh R., Rao S.G., Kondaiah P. (2011). Role of TGF-beta and BMP7 in the pathogenesis of oral submucous fibrosis. Growth Factors.

[40] Bartkova J., Horejsi Z., Koed K., Kramer A., Tort F., Zieger K., Guldberg P., Sehested M., Nesland J.M., Lukas C. (2005). DNA damage response as a candidate anti-cancer barrier in early human tumorigenesis. Nature.

[41] Gorgoulis V.G., Vassiliou L.V., Karakaidos P., Zacharatos P., Kotsinas A., Liloglou T., Venere M., Ditullio R.A., Kastrinakis N.G., Levy B. (2005). Activation of the DNA damage checkpoint and genomic instability in human precancerous lesions. Nature.

[42] Bergink S., Jentsch S. (2009). Principles of ubiquitin and SUMO modifications in DNA repair. Nature.

[43] Sugasawa K., Okuda Y., Saijo M., Nishi R., Matsuda N., Chu G., Mori T., Iwai S., Tanaka K., Hanaoka F. (2005). UV-induced ubiquitylation of XPC protein mediated by UV-DDB-ubiquitin ligase complex. Cell.

[44] Leemans C.R., Braakhuis B.J., Brakenhoff R.H. (2011). The molecular biology of head and neck cancer. Nat. Rev. Cancer.

[45] Argiris A., Karamouzis M.V., Raben D., Ferris R.L. (2008). Head and neck cancer. Lancet.

[46] Ang K.K., Harris J., Wheeler R., Weber R., Rosenthal D.I., Nguyen-Tan P.F., Westra W.H., Chung C.H., Jordan R.C., Lu C. (2010). Human papillomavirus and survival of patients with oropharyngeal cancer. N. Engl. J. Med..

[47] Networks T.C.G.A. (2015). Comprehensive genomic characterization of head and neck squamous cell carcinomas. Nature.

[48] Smeets S.J., van der Plas M., Schaaij-Visser T.B., van Veen E.A., van Meerloo J., Braakhuis B.J., Steenbergen R.D., Brakenhoff R.H. (2011). Immortalization of oral keratinocytes by functional inactivation of the p53 and pRb pathways. Int. J. Cancer.

[49] Zhou G., Liu Z., Myers J.N. (2016). TP53 Mutations in Head and Neck Squamous Cell Carcinoma and Their Impact on Disease Progression and Treatment Response. J. Cell. Biochem..

[50] Gu B., Zhu W.G. (2012). Surf the post-translational modification network of p53 regulation. Int. J. Biol. Sci..

[51] Sykes S.M., Mellert H.S., Holbert M.A., Li K., Marmorstein R., Lane W.S., McMahon S.B. (2006). Acetylation of the p53 DNA-binding domain regulates apoptosis induction. Mol. Cell.

[52] Arbely E., Natan E., Brandt T., Allen M.D., Veprintsev D.B., Robinson C.V., Chin J.W., Joerger A.C., Fersht A.R. (2011). Acetylation of lysine 120 of p53 endows DNA-binding specificity at effective physiological salt concentration. Proc. Natl. Acad. Sci. USA.

[53] Zhang H., Somasundaram K., Peng Y., Tian H., Zhang H., Bi D., Weber B.L., El-Deiry W.S. (1998). BRCA1 physically associates with p53 and stimulates its transcriptional activity. Oncogene.

[54] Takimoto R., MacLachlan T.K., Dicker D.T., Niitsu Y., Mori T., el-Deiry W.S. (2002). BRCA1 transcriptionally regulates damaged DNA binding protein (DDB2) in the DNA repair response following UV-irradiation. Cancer Biol..

[55] MacLachlan T.K., Takimoto R., El-Deiry W.S. (2002). BRCA1 directs a selective p53-dependent transcriptional response towards growth arrest and DNA repair targets. Mol. Cell. Biol..

[56] Chiang S.L., Jiang S.S., Wang Y.J., Chiang H.C., Chen P.H., Tu H.P., Ho K.Y., Tsai Y.S., Chang I.S., Ko Y.C. (2007). Characterization of arecoline-induced effects on cytotoxicity in normal human gingival fibroblasts by global gene expression profiling. Toxicol. Sci..

[57] Choudhury Y., Sharan R.N. (2009). Altered p53 response and enhanced transgenerational transmission of carcinogenic risk upon exposure of mice to betel nut. Environ. Toxicol. Pharmacol..

[58] Choudhury Y., Sharan R.N. (2011). Altered BRCA1 and BRCA2 responses and mutation of BRCA1 gene in mice exposed chronically and transgenerationally to aqueous extract of betel nut (AEBN). Environ. Toxicol. Pharmacol..

[59] Miyashita H., Mori S., Tanda N., Nakayama K., Kanzaki A., Sato A., Morikawa H., Motegi K., Takebayashi Y., Fukumoto M. (2001). Loss of heterozygosity of nucleotide excision repair factors in sporadic oral squamous cell carcinoma using microdissected tissue. Oncol. Rep..

[60] Knijnenburg T.A., Wang L., Zimmermann M.T., Chambwe N., Gao G.F., Cherniack A.D., Fan H., Shen H., Way G.P., Greene C.S. (2018). Genomic and Molecular Landscape of DNA Damage Repair Deficiency across The Cancer Genome Atlas. Cell Rep..

[61] Chou S.T., Peng H.Y., Mo K.C., Hsu Y.M., Wu G.H., Hsiao J.R., Lin S.F., Wang H.D., Shiah S.G. (2019). MicroRNA-486–3p functions as a tumor suppressor in oral cancer by targeting DDR1. J. Exp. Clin. Cancer Res. Cr.

[62] Shiah S.G., Hsiao J.R., Chang H.J., Hsu Y.M., Wu G.H., Peng H.Y., Chou S.T., Kuo C.C., Chang J.Y. (2020). MiR-30a and miR-379 modulate retinoic acid pathway by targeting DNA methyltransferase 3B in oral cancer. J. Biomed. Sci..

[63] Yang H., Liu J., Jing J., Wang Z., Li Y., Gou K., Feng X., Yuan Y., Xing C. (2018). Expression of DDB2 Protein in the Initiation, Progression, and Prognosis of Colorectal Cancer. Dig. Dis. Sci..

[64] De Sousa J.F., Torrieri R., Serafim R.B., Di Cristofaro L.F., Escanfella F.D., Ribeiro R., Zanette D.L., Paco-Larson M.L., da Silva W.A., Tirapelli D.P. (2017). Expression signatures of DNA repair genes correlate with survival prognosis of astrocytoma patients. Tumour Biol. J. Int. Soc. Oncodev. Biol. Med..

[65] Bommi P.V., Ravindran S., Raychaudhuri P., Bagchi S. (2018). DDB2 regulates Epithelial-to-Mesenchymal Transition (EMT) in Oral/Head and Neck Squamous Cell Carcinoma. Oncotarget.

[66] Roy N., Bommi P.V., Bhat U.G., Bhattacharjee S., Elangovan I., Li J., Patra K.C., Kopanja D., Blunier A., Benya R. (2013). DDB2 suppresses epithelial-to-mesenchymal transition in colon cancer. Cancer Res..

[67] Van Staalduinen J., Baker D., Ten Dijke P., van Dam H. (2018). Epithelial-mesenchymal-transition-inducing transcription factors: New targets for tackling chemoresistance in cancer?. Oncogene.

[68] Ennen M., Klotz R., Touche N., Pinel S., Barbieux C., Besancenot V., Brunner E., Thiebaut D., Jung A.C., Ledrappier S. (2013). DDB2: A novel regulator of NF-kappaB and breast tumor invasion. Cancer Res..

[69] Chang Y.C., Tsai C.H., Lai Y.L., Yu C.C., Chi W.Y., Li J.J., Chang W.W. (2014). Arecoline-induced myofibroblast transdifferentiation from human buccal mucosal fibroblasts is mediated by ZEB1. J. Cell. Mol. Med..

[70] Wang T.Y., Peng C.Y., Lee S.S., Chou M.Y., Yu C.C., Chang Y.C. (2016). Acquisition cancer stemness, mesenchymal transdifferentiation, and chemoresistance properties by chronic exposure of oral epithelial cells to arecoline. Oncotarget.

[71] Zheng L., Jian X., Guo F., Li N., Jiang C., Yin P., Min A.J., Huang L. (2015). miR-203 inhibits arecoline-induced epithelial-mesenchymal transition by regulating secreted frizzled-related protein 4 and transmembrane-4 L six family member 1 in oral submucous fibrosis. Oncol. Rep..

[72] Lin C.S., Wang Y.C., Huang J.L., Hung C.C., Chen J.Y. (2012). Autophagy and reactive oxygen species modulate cytotoxicity induced by suppression of ATM kinase activity in head and neck cancer cells. Oral Oncol..

[73] Nair J., Ohshima H., Friesen M., Croisy A., Bhide S.V., Bartsch H. (1985). Tobacco-specific and betel nut-specific N-nitroso compounds: Occurrence in saliva and urine of betel quid chewers and formation in vitro by nitrosation of betel quid. Carcinogenesis.

[74] Lin C.S., Chiou W.Y., Lee K.W., Chen T.F., Lin Y.J., Huang J.L. (2016). Xeroderma pigmentosum, complementation group D expression in H1299 lung cancer cells following benzo[a]pyrene exposure as well as in head and neck cancer patients. J. Toxicol. Environ. Health A.

[75] Kuo K.K., Lee K.T., Chen K.K., Yang Y.H., Lin Y.C., Tsai M.H., Wuputra K., Lee Y.L., Ku C.C., Miyoshi H. (2016). Positive Feedback Loop of OCT4 and c-JUN Expedites Cancer Stemness in Liver Cancer. Stem Cells.

[76] Donner A.J., Szostek S., Hoover J.M., Espinosa J.M. (2007). CDK8 is a stimulus-specific positive coregulator of p53 target genes. Mol. Cell.

[77] Lee K.W., Tsai Y.S., Chiang F.Y., Huang J.L., Ho K.Y., Yang Y.H., Kuo W.R., Chen M.K., Lin C.S. (2011). Lower ataxia telangiectasia mutated (ATM) mRNA expression is correlated with poor outcome of laryngeal and pharyngeal cancer patients. Ann. Oncol..

