# Supplementary material for: Downregulation of the DNA Repair Gene DDB2 by Arecoline Is through p53’s DNA-Binding Domain and Is Correlated with Poor Outcome of Head and Neck Cancer Patients with Betel Quid Consumption
Source: Cancers (Basel). 2020 Jul 25;12(8):2053. doi: 10.3390/cancers12082053 (PMC7465463; doi:10.3390/cancers12082053)
Supplement: Supplementary file 1 [file cancers-12-02053-s001.pdf]

Supplementary Materials

Downregulation of the DNA Repair Gene DDB2 by Arecoline Is through p53′s DNA-Binding Domain and Is Correlated with Poor Outcome of Head and Neck Cancer Patients with Betel Quid Consumption

Yu-Chu Wang, Jau-Ling Huang, Ka-Wo Lee, Hsing-Han Lu, Yuan-Jen Lin, Long-Fong Chen, Chung-Sheng Wang, Yun-Chiao Cheng, Zih-Ting Zeng, Pei-Yi Chu and Chang-Shen Lin

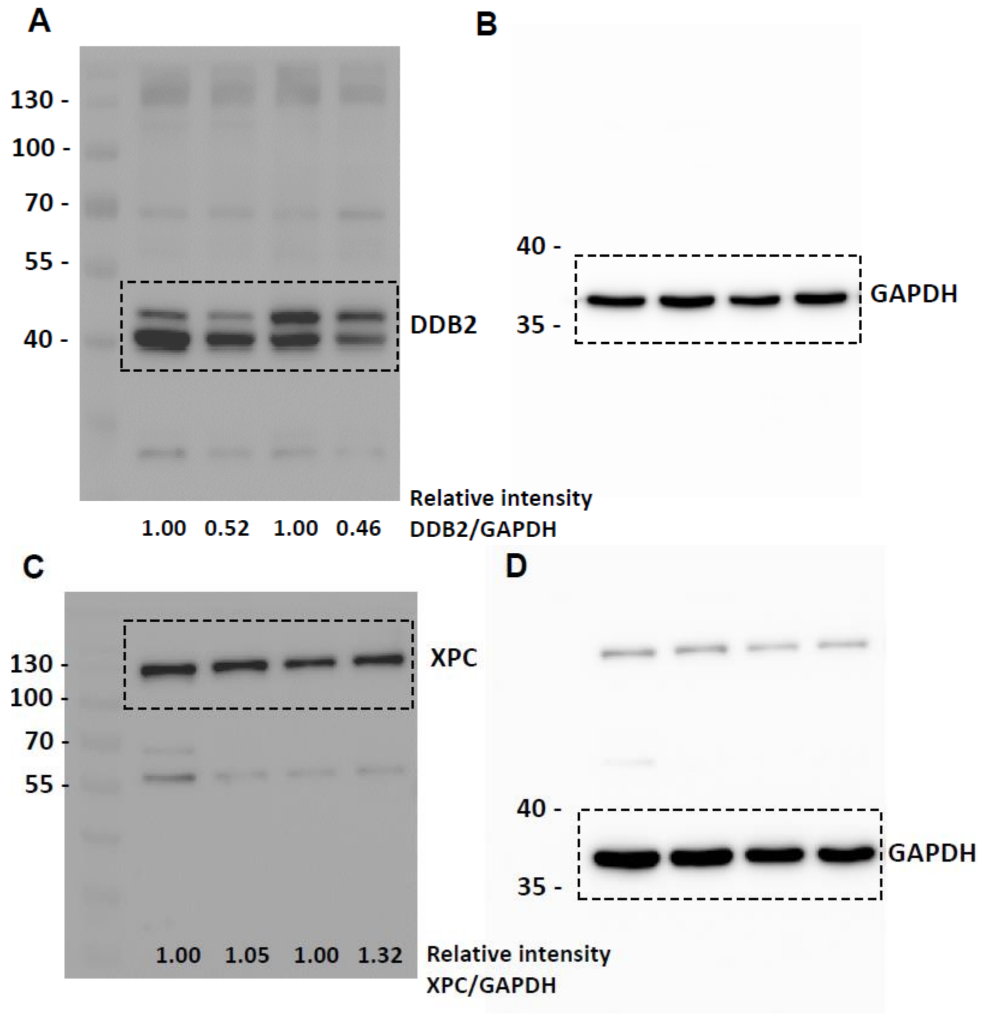

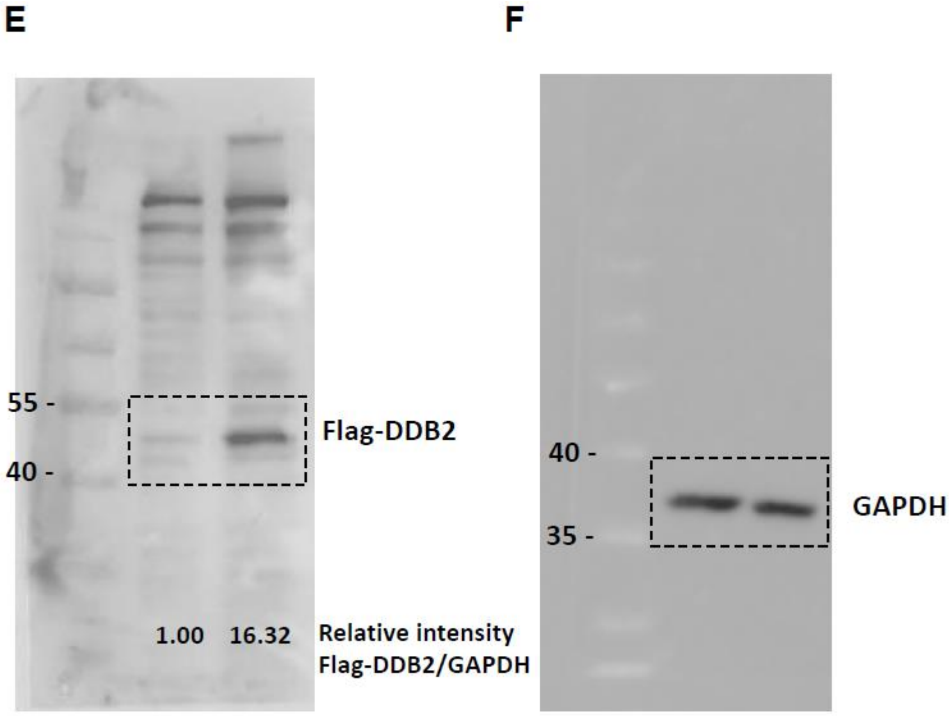

Figure S1. The full-length blots for Figures 1D and 1F. The blots of DDB2 **(A)** and its loading control GAPDH **(B)** for Figure 1D. The blots of XPC **(C)** and its loading control GAPDH **(D)** for Figure 1D. The blots of DDB2 **(E)** and its loading control GAPDH **(F)** for Figure 1F. Panels A, C, E, and F are superimposed images of chemiluminescence (target proteins) and visual light (marker) and thus exhibit with high image backgrounds.

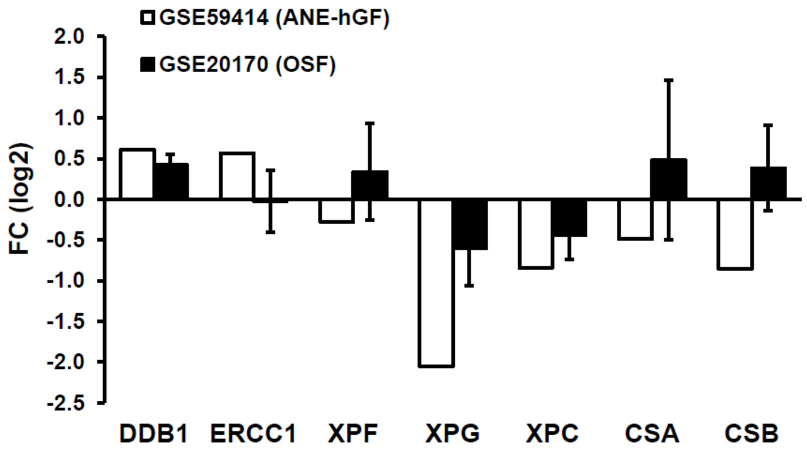

Figure S2. Expression of NER genes in GSE59414, which is shown by an average of 2 samples of areca nut extract (ANE)-treated human gingival fibroblast (hGF), and in GSE20170, which is shown by an average of 10 oral submucous fibroblast (OSF) and standard deviations. FC (log2), fold-changed with log2-transformed.

© 2020 by the authors. Licensee MDPI, Basel, Switzerland. This article is an open access article distributed under the terms and conditions of the Creative Commons Attribution (CC BY) license (http://creativecommons.org/licenses/by/4.0/).
